# Surviving Salt: How Do Extremophiles Do It?

**DOI:** 10.1371/journal.pbio.1000258

**Published:** 2009-12-15

**Authors:** Mary Hoff

**Affiliations:** Freelance Science Writer, Stillwater, Minnesota, United States of America

**Figure pbio-1000258-g001:**
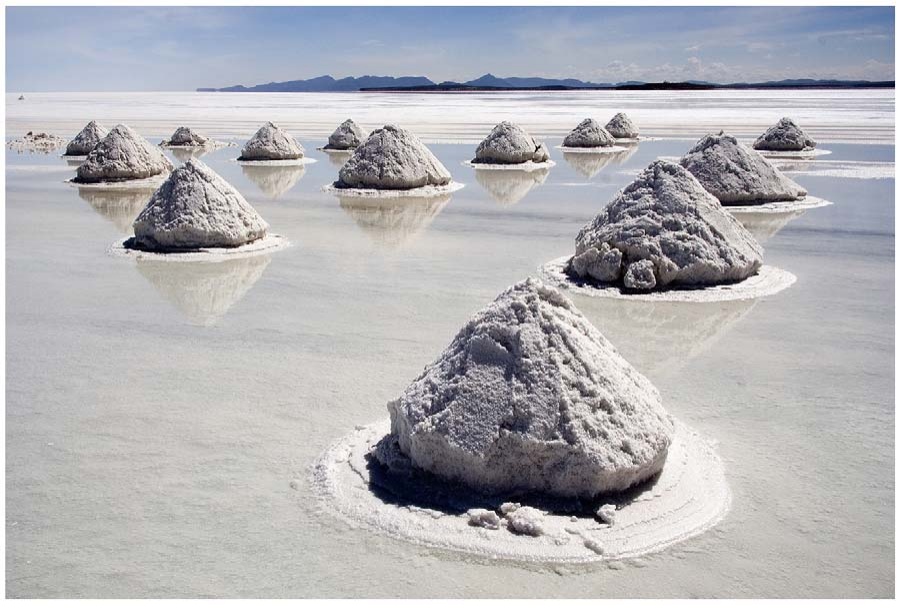
Halophilic archaea thrive in harsh environments like the saline shown here, on the Salar de Uyuni (Bolivia). At the molecular level, proteins that belong to these extremophiles have evolved toward a biased amino acid composition, which reduces the interactions with the solvent. (Image Credit: Luca Galuzzi, http://www.galuzzi.it).

[Fig pbio-1000258-g001]Immersed in waters saltier than chicken soup, salt-tolerant “halophilic” microorganisms are able to thrive in conditions that would reduce a less-adapted organism to a shriveled remnant. One way halophilic archaea avoid this fate is by bathing their molecular machinery in a similarly salty intracellular environment that would cause ordinary proteins to lose their shape. How do the proteins inside these cells survive?

At least part of the answer seems to relate to an abundance of certain amino acid residues on the protein surface. Salt-tolerant proteins tend to have lots of aspartic acid, glutamic acid, and other non-hydrophobic residues on their surfaces. They also tend to have fewer lysines than similar proteins from non-halophilic counterparts, their places often being taken by bulkier arginine instead. What traits of these residues make them salt-friendly? One school of thought suggests it's the charge they carry. Another suggests it's not so much charge as the size of the side chain that gives these residues their evolutionary edge in a saline setting.

To determine what allows extremophile proteins to tolerate salt, Oscar Millet, Xavier Tadeo, and colleagues analyzed the structure and thermodynamics of three protein domains under different salt concentrations: *Hv* 1ALigNm from *Haloferax volcanii*, a halophile found in places like the Dead Sea and Great Salt Lake; *Ec* 1ALigN from *Escherichia coli*; and ProtL from the mesophilic *Streptococcus magnus*. They then used site-directed mutagenesis to create three broad classes of domain mutations: mutations in which the amino acid side chain length changed but the charge did not, mutations in which the charge changed but the length did not, and mutations in which both length and charge were altered.

Analyzing the structure and thermodyamics of the altered domains, the investigators found that reducing the size of the residue's side chain without changing its charge improved salt tolerance in both *Hv* 1ALigN and *Ec* 1ALigN. Lengthening the side chain had the opposite impact on mutation of all three domains being investigated. Mutations increasing the negative charge of the domain, on the other hand, showed little impact on salt tolerance in any of the three protein domains studied.

The researchers also investigated whether the tendency of halophiles' protein surfaces to have arginine rather than lysine had any effect on the proteins' ability to cope with salt. They found that substituting lysines for arginines or other polar residues with small side chains increased the salt tolerance of ProtL. Introduction of lysine residues, with their long side chains onto the surface of *Hv* 1ALigN, decreased stability in salty solutions.

To further explore the connections between residue side chains and salt tolerance, the researchers used other types of mutagenic systems to alter a different set of residues on the surface of the proteins. The results indicated that it is the nature of a mutation, and not its location, that alters the proteins' ability to withstand a salt assault.

Interestingly, the researchers also discovered that salt tolerance is not the only characteristic conferred by the particular mix of residues found in halophilic proteins. Their residue substitution experiments also showed that the abundance of aspartic acid and glutamic acid characteristics of halophilic proteins is good not only for salt tolerance but also for solubility—another valuable trait in conditions typical of high-salt environments.

Concluding from their experiments that residue compactness and not charge is what matters most when it comes to surviving in salt, the researchers assessed salt tolerance and (using high-resolution NMR) the accessible solvent surface area in two multiply mutated versions of ProtL, in an attempt to quantify the relationship. They found that a decrease in surface area correlated well with an increase in salt tolerance, indicating that tight packing is the trick for preventing salt from trashing proteins. At the same time, they also discovered that such packing is not a survival skill that's fit for all conditions. When the same mutants were exposed to a low-salt environment, the molecules destabilized, indicating that the mutations that made them better able to tolerate salt also made them less able to tolerate its absence. All together, these findings not only shed light on a fascinating evolutionary trait, but also provide valuable insights into what it takes to survive in a saline environment—information that could be applied to engineer proteins in a way that does not alter their biological function but does confer salt tolerance beyond what they naturally would endure.

**Tadeo X, López-Méndez B, Trigueros T, Laín A, Castaño D, et al. (2009) Structural Basis for the Aminoacid Composition of Proteins from Halophilic Archea. doi:10.1371/journal.pbio.1000257**

